# PERCEIVED WORK ENVIRONMENT AND OUTCOMES AMONG CARE STAFF IN LONG-TERM CARE SETTINGS DURING COVID-19

**DOI:** 10.1093/geroni/igad104.2853

**Published:** 2023-12-21

**Authors:** Eunhee Cho, Min Jung Kim, Kyung Hee Lee, Bada Kang, Jiyoon Jang, Jinhee Shin, Sameh Eltaybani, Noriko Yamamoto-Mitani

**Affiliations:** Yonsei University College of Nursing, Seoul, Republic of Korea; Yonsei University, Seoul, Republic of Korea; Yonsei University, Seoul, Republic of Korea; Yonsei University, Seoul, Republic of Korea; Yonsei University, Seoul, Republic of Korea; Woosuk University, Jeonju, Republic of Korea; The University of Tokyo, Tokyo, Tokyo, Japan; The University of Tokyo, Tokyo, Tokyo, Japan

## Abstract

Coronavirus disease (COVID-19) has severely affected residents in long-term care (LTC) facilities. However, how care staff in LTC facilities perceive their work environment during the pandemic and its potential impact on their work outcome are less understood. This study examined the associations between perceived work environment, staffing levels, and educational status of care staff and their general and COVID-19 specific work outcomes during the pandemic in LTC facilities. The study employed a cross-sectional, observational, correlational design. A total of 207 care staff were conveniently recruited from 30 LTC facilities in South Korea. The perceived work environment, staffing levels, educational status of care staff, and their work outcomes were collected using questionnaires. Multivariable binary logistic regressions were conducted, controlling for the characteristics of care staff and facilities included. Approximately 45% of the participants were either nurse aids or care workers, and 38% were registered nurses. Nearly half reported that their work environment was poor. Care staff who perceived their work environment to be poor were more likely to be dissatisfied with their work (odds ratio [OR] 15.40), experience high burnout (OR6.93), intend to leave the facility within a year (OR7.83), and experience increased overtime work due to COVID-19 (OR4.03). They were also less likely to perceive an immediate response to COVID-19 from their facility (OR0.45). The LTC work environment should be improved from its current state and government-led initiatives for infection control should be implemented to enable a better response to future public health crises in LTC settings.

